# Slanted versus Augmented Recession for Horizontal Strabismus

**DOI:** 10.18502/jovr.v14i4.5453

**Published:** 2019-10-24

**Authors:** Zhale Rajavi, Mohadeseh Feizi, Sayed Aliasghar Nabavi, Hamideh Sabbaghi, Narges Behradfar, Mehdi Yaseri, Mohammad Faghihi, Saeid Abdi

**Affiliations:** ^1^Ophthalmic Epidemiology Research Center, Shahid Beheshti University of Medical Sciences, Tehran, Iran; ^2^Department of Ophthalmology, Torfeh Hospital, Shahid Beheshti University of Medical Sciences, Tehran, Iran; ^3^Negah Specialty Ophthalmic Research Center, Shahid Beheshti University of Medical Sciences, Tehran, Iran; ^4^Ophthalmic Research Center, Shahid Beheshti University of Medical Sciences, Tehran, Iran; ^5^Department of Optometry, School of Rehabilitation, Shahid Beheshti University of Medical Sciences, Tehran, Iran; ^6^Department of Epidemiology and Biostatistics, Tehran University of Medical Sciences, Tehran, Iran

**Keywords:** Accommodative Convergence to Accommodation Ratio, Augmented Recession, Horizontal Strabismus, Slanted Recession

## Abstract

**Purpose:**

To compare the surgical outcomes of slanted versus augmented recession in patients with horizontal strabismus.

**Methods:**

In this randomized clinical trial, a total of 100 esotropic (ET) and exotropic (XT) patients with a high AC/A ratio which was defined as a difference of ≥ 10 prism diopters (pd) between the distance and near deviations were included if the patients had a distance deviation ≥ 15 pd. Patients were randomly assigned into the slanted (*n* = 26 in ET and *n* = 24 in XT group) and augmented recession groups (*n* = 25 in ET and *n* = 25 in XT group). In the slanted group, recession was performed on the superior and inferior poles of the muscle based on the distance and near deviations, respectively, while in the augmented recession group, the muscles were recessed 1.00 or 1.50 mm more than the standard amount according to the distance and near difference between 10 and 20 pd or > 20 pd, respectively.

**Results:**

The mean age was 9.8 ± 9.6 years and 63% were female. There was a significant postoperative reduction of difference in convergence excess in ET cases compared to patients who underwent the augmented recession procedure (12.65 ± 6.16 vs 8.64 ± 6.1 pd, *P* = 0.014). Among our XT groups, there was no significant difference in postoperative reduction in the XT angle in the slanted group compared with the augmented group (*P* > 0.05).

**Conclusion:**

Slanted recession is recommended in convergence excess ET patients. In XT patients, either slanted or augmented recession may be chosen according to the priority and experience of the surgeon.

##  INTRODUCTION

Exotropic (XT) and esotropic (ET) patients with abnormal accommodative convergence to accommodation (AC/A) ratio present a difference between distance and near deviations which are manifested even after correction of refractive errors and achievement of the best corrected visual acuity (BCVA).^[1,2]^

According to the literature, the main cause of this difference in ET cases with a high AC/A ratio could be attributed to the excessive accommodative convergence with an insufficient fusional divergence at near distance of fixation.^[3]^ In XT patients with a high AC/A ratio, near deviation is increased with bilateral +3.00 D lenses immediately after patching; otherwise, patients have true divergence excess (DE). In both conditions, the possibility of esotropia at near may be present postoperatively. In addition, in XT patients with a low AC/A ratio, the weak fusional convergence and reduced accommodative amplitude have been reported as possible etiological factors of convergence insufficiency (CI).^[1,4]^ Moreover, in XT patients with CI, surgery for distance deviation would show near under correction postoperatively.

Surgery is indicated in cases which do not respond to the non-surgical treatments including refractive error correction, orthoptic modalities, prism, and also in patients with distance deviation of ≥ 15 pd.^[5,6]^ Slanted or augmented bilateral lateral rectus recession (LRRec) or bilateral medial rectus recession (MRRec) or new R & R (LRRec for distance and MRRec for near deviations) are suggested methods for these patients with a success rate of 69–92% in different studies.^[1,2][7][8][9][10]^

ET patients with less near deviation (low AC/A ratio) are rare, and there is not a consensus regarding the unique surgical procedure for these patients.^[11]^

Muscle slanted recession method seems more logical and is easier with fewer side effects compared to the other procedures such as posterior fixation suture and can be applied in both ET and XT patients.^[1,2][7][8][9][10]^ Therefore, we aimed to compare the surgical outcomes of slanted and augmented recession methods on patients with horizontal strabismus having a different angle of deviation at distance and near positions with abnormal AC/A ratio either low or high.

##  METHODS

This randomized clinical trial was performed on 100 patients with horizontal deviation through sequential selection. All constant or intermittent ET and XT patients with abnormal AC/A ratio (defined as a difference of ≤ 10 pd between distance and near deviations) were enrolled, if the patients had a distance deviation ≥ 15 pd. This study was approved by the Ethics Committee of the Ophthalmic Research Center, Shahid Beheshti University of Medical Sciences, Tehran, Iran and was registered at https://clinicaltrials.gov (NCT03555045).

Patients with simultaneous horizontal and vertical deviation, history of extraocular muscle surgery, extraocular muscle palsy, fixation instability (nystagmus, eccentric fixation), restrictive strabismus, orbital anomalies, mental retardation, general and ophthalmic disorders, simulated DE, subjects less than five years old and those with poor cooperation were excluded from this study.

After patients or their parents signed informed consent, questions regarding demographic characteristics such as the age at the time of operation, gender, parent consanguinity, family history of strabismus, and prematurity were asked and recorded in their files.

###  Ocular and Visual Examinations

The comprehensive ophthalmic examination including cyclorefraction (45 min after instillation of tropicamide 1% and cyclopentolate 1%), assessment of BCVA, and evaluation of extraocular muscle motility including version and duction were performed. Next, ocular deviation was measured at both distant (6 m) and near (33 cm) using an alternate prism cover test. For accurate fixation, all patients were asked to fixate on an optotype of the Snellen visual acuity chart sized one or two lines better than their BCVA for distance deviation measurement and an accommodative target for near deviation measurement. A- or V-pattern was also determined if the difference of deviation between 30° superior and inferior of primary position was 10 or 15 pd, respectively.^[12,13]^ Stereopsis was measured using a Titmus test at the near position. Ocular anterior and posterior segments were examined using slit lamp and indirect ophthalmoscopy.

###  Definitions

Amblyopia was considered if the BCVA was equal or worse than 0.30 LogMAR in one eye or two BCVA lines of difference between the two eyes.

Stereopsis was classified into three groups of central (≤ 100 sec/arc), peripheral (100–3000 sec/arc), and suppression (≥ 3000 or not having any binocular depth perception).

Convergence excess (CE) was considered in ET patients with more deviation (≥ 10 pd) at near compared to the distance after refractive error correction.

Simulated DE was considered if tenacious proximal fusion (TPF) was broken in an XT patient with more deviation at near after patching of one eye for 60 min.

High AC/A ratio in ET patients was considered with more deviation (≥ 10 pd) at near compared to distance after refractive error correction (CE).

High AC/A ratio was considered if near deviation was not increased with monocular patching in XT patients, but it was increased after wearing of +3.00D lenses. Therefore, high AC/A ratio was detected; otherwise the diagnosis of DE was considered.^[14]^

CI was considered in cases that had higher exotropia at near compared with distance deviation (at least a difference of 10 pd) after refractive error correction.

###  Surgical Procedure

###  General points

ET and XT patients were studied in the two groups (slanted and augmented recession), and the procedures were performed on medial or lateral rectus muscles according to the deviation type.

After limbal incision of conjunctiva and tendon, the selected rectus muscle was hooked, dissected, and sutured with 6-0 VicrylⓇ (polyglactin 910, coated VicrylⓇ, Ethicon, Blue Ash, OH, USA), then the muscle was disinserted and resutured to the sclera posteriorly according to the Park's table.^[15]^

###  Slanted recession method

In the slanted groups, the superior pole of the rectus muscles was recessed for distance deviation, and the inferior pole of the same rectus muscle was recessed for near deviation in both eyes. The amount of recession was considered based on the Park's table.^[15]^

The amount of slant was defined as the absolute difference of superior and inferior recession of the medial or lateral rectus muscles.

###  Augmented recession method

In the augmented recession group, the patients were operated bilaterally according to the distance measurement and based on the difference between distance and near deviations from 10 to 20 pd, 1 mm, and > 20 pd; 1.5 mm was added to or subtracted from the distance deviation as suggested by Park's table.^[15]^ For instance, in patients with ET = 20 and ET' = 35, bilateral LRRec was performed for ET = 20 (4) + 1 = 5 mm. In patients with XT = 20 and XT' = 35, bilateral LRRec was performed for XT = 20 (5) + 1 = 6 mm. In patients with XT = 30 and XT' = 15 (either high AC/A ratio or true DE), bilateral LRRec was performed for XT = 30 (7) – 1 = 6 mm.

Follow-up visits were performed on the first day, the first week, and the first, third, sixth, and twelfth months after the surgery. The results of patients who had at least three months of follow-up were analyzed. Postoperative distance and near deviations were measured in the same way as the preoperative method. Postoperative distance and near deviations < 10 pd were considered successful outcomes in each group.

In addition, dose-response in both groups were calculated based on the following formula:

Dose response = | preoperative  far  and  near  difference -- postoperative  far  and  near  difference | amount  of  slant  or  recession  Dose response = or | reduction | amount  of  slant  or  recession 

For instance, a patient with an XT = 30 at far and XT' = 40 at near, a bilateral LRRec was performed 7 mm at the superior and 8 mm at the inferior poles of the muscle. Regarding the calculation of dose-response, this value was used in the aforementioned formula, and two folds of slant were calculated for the analysis (2 × 1).

###  Sample Size

To have a power of 95% to detect a difference of 5 pd in transparency between the two groups, 25 samples in each group were required. In this calculation, the standard deviation of the transparency was assumed to be 5 in both groups, and the type I error was set at 0.05.

###  Randomization

Using a computer program, a biostatistician (MY) generated the sequence of patients' assignment with a permuted-block randomization method. The block length varied from two to six. The randomization sequence was concealed from the investigators. Randomization was separately performed for ET and XT groups.

###  Statistical Analysis

To present data, mean, standard deviation, median, and range were used. To evaluate the normal distribution of data, we used a Kolmogorov–Smirnov test and Q-Q plot. To compare the groups, a *t*-test and a Mann–Whitney U-test were used. Additionally, we used a Wilcoxon signed-rank test to assess changes within the groups. Relationships of the different variables were assessed using Spearman's Rank correlation coefficient. Furthermore, the interaction analysis within the linear regression model was applied to evaluate the difference of esotropia and exotropia with regard to the inferiority of the slanted surgical technique. All statistical methods were performed with SPSS software (SPSS Statistics for Windows version 23.0, IBM Corporation, Armonk, NY, USA). All tests were two-sided, and *p* < 0.05 was considered statistically significant.

##  RESULTS

The present study was conducted on 100 patients with different horizontal deviation at distance and near ≥10 pd; 50 ET and 50 XT patients randomly underwent surgery by the slanted or augmented recession technique, respectively. In this study, the mean surgical age was 9.83 ± 9.65 years old, and 63% of the study subjects were female.

Table 1 presents the baseline characteristics of our study population in both ET and XT groups. Although more study patients were female, the difference was not statistically significant between the slanted and augmented recession groups. In addition, there was no difference in these groups regarding other baseline characteristics, including age, prematurity, parental consanguinity, and family history of strabismus.

All study subjects underwent follow-ups for at least three months with an average of 10 ± 9 months; 84% (*n* = 42) of ET and 60% (*n* = 30) of XT patients had a minimum follow-up of six months.

Table 2 shows the clinical characteristics of slanted and augmented recession in both ET and XT patients. Amblyopia was detected in 13.4% (*n* = 13) of the study population, and it was found in 20% (*n* = 10) of ET and 6% (*n* = 3) of XT patients. As seen, 27% of patients presented ≤ 100 sec/arc stereopsis after the surgery.

Preoperatively, in the ET group, A- and V-patterns as well as overaction of the inferior oblique muscle (IOOA) were observed in 2%, 41%, and 45% of patients respectively, while more XT patients had A-pattern (12%) and fewer showed V-pattern (18%) and IOOA (10%). Postoperatively, 30.8 % of the slanted ET cases had V-pattern, and no cases had A-pattern. In the slanted XT group, the percentages of A- and V-pattern were equal (8%).

In the present study, all ET patients had CE esotropia with a high AC/A ratio. As Table 3 shows, although there was not any significant difference in the distance and near disparity between the slanted and augmented recession groups preoperatively, statistically significant reduction was found in the slanted recession group compared with the augmented group postoperatively (12.65 vs 8.64, *P *= 0.014). The mean slant was 2.38 ± 1.33 mm (range, 1 to 5 mm) for ET patients and 1.48 ± 0.79 mm

(range, 0.5 to 3 mm) for XT patients, and the mean augmentation was 1.125 ± 0.25 mm for ET and 1.02 ± 0.25 mm for XT patients. A 58% success rate was obtained in the slanted group and a 28% success rate was observed in the augmented recession group.

As Table 4 shows, 29 XT patients had CI and 20 patients had true DE or exotropia with a high AC/A ratio. There was no significant difference between the slanted and augmented recession groups regarding distance and near disparity, preoperatively. The mean slant for the esotropia, exotropia, and augmented recession groups was 2.38 ± 1.33 mm, 1.48 ± 0.79 mm, and 1.00–1.50 mm, respectively. Similar findings were also obtained in an analysis of each XT subgroup.

In a comparison of the slanted technique between the ET and XT patients, it was revealed that the difference of reduction was greater in ET cases compared to the XT cases (12.65 Table 3 – 8.46 Table 4 = 4.2 pd, *P *= 0.04).

There were no surgical complications such as postoperative diplopia, overcorrection, ocular torsion, and new cases of A–V patterns in our study groups.

**Table 1:** Baseline characteristics of slanted and augmented recession in both eso- and exotropic patients


**Factors**	**Total**	**Groups**					
		**ET**			**XT**		
		**Slanted**	**Augmented **	***P*-value**	**Slanted**	**Augmented **	***P*-value**
N (%)	100	26 (26%)	24 (24%)	25 (25%)	25 (25%)	
Age of operation (years)	Mean ± SD	9.83 ± 9.65	9.6 ± 9.84	7.64 ± 2.33	0.333†	11.21 ± 8.31	12.92 ± 13.48	0.449†
Sex	M	37 (37.0%)	10 (38.5%)	10 (40.0%)	> 0.999*	8 (33.3%)	9 (36.0%)	0.834*
	F	63 (63.0%)	16 (61.5%)	15 (60.0%)	16 (66.7%)	16 (64.0%)	
Prematurity	No	97 (97.0%)	26 (100.0%)	23 (92.0%)	0.054**	23 (95.8%)	25 (100.0%)	0.237**
	Yes	3 (3.0%)	0 (0.0%)	2 (8.0%)	1 (4.2%)	0 (0.0%)	
Parent consanguinity	No	84 (84.0%)	19 (73.1%)	19 (76.0%)	0.822*	23 (95.8%)	23 (92.0%)	0.678**
	Yes	16 (16.0%)	7 (26.9%)	6 (24.0%)	1 (4.2%)	2 (8.0%)	
Family H/O strabismus	No	88 (88.0%)	22 (84.6%)	21 (84.0%)	> 0.999**	21 (87.5%)	24 (96.0%)	0.155**
	Yes	12 (12.0%)	4 (15.4%)	4 (16.0%)	3 (12.5%)	1 (4.0%)	
	
	
ET, esotropia; XT, exotropia; N, number; FU, follow-up; H/O, history of; M, male; F, female; P, probability								
†Based on t-test								
*Based on Chi-Square test								
**Based on Fisher exact test								

**Table 2:** Clinical characteristics of slanted and augmented recession in both eso- and exotropic patients


**Factors**	**Total**	**Groups**					
		**ET**			**XT**		
		**Slanted**	**Augmented**	***P*-value**	**Slanted**	**Augmented**	***P*-value**
Pre-op. SE (D)	Mean ± SD	1.73 ± 2.51	1.29 ± 2.71	2.19 ± 2.23	0.070‡	0.36 ± 1.69	0.30 ± 1.37	0.849‡
Pre-op. BCVA (LogMAR)	Mean ± SD	0.19 ± 0.21	0.14 ± 0.13	0.497‡	0.10 ± 0.14	0.11 ± 0.11	0.310‡
Post-op. BCVA (LogMAR)	Mean ± SD	0.12 ± 0.16	0.17 ± 0.2	0.07 ± 0.09	0.002‡	0.07 ± 0.12	0.09 ± 0.09	0.611‡
Amblyopia (%)	No	84 (86.6%)	18 (69.2%)	23 (92.0%)	0.075*	21 (91.3%)	22 (95.7%)	> 0.99*
	Yes	13 (13.4%)	8 (30.8%)	2 (8.0%)	2 (8.7%)	1 (4.3%)	
Pre-op. Stereopsis (%, sec/arc)	Central (≤ 100)	15 (15%)	1 (3.8%)	0 (0%)	0.477‡	6 (25%)	8 (32%)	0.87‡
	Peripheral (100 to 3000)	15 (15.4%)	1 (3.8%)	3 (12%)	6 (25%)	5 (20%)	
	Suppression (≥ 3000)	70 (70%)	24 (92.3%)	22 (88%)	12 (50%)	12 (48%)	
Post-op. Stereopsis (%, sec/arc)	Central (≤ 100)	27 (27%)	2 (7.7%)	4 (16%)	0.516‡	11 (45.8%)	10 (40%)	0.578‡
	Peripheral (100 to 3000)	31 (31%)	7 (26.9%)	8 (32%)	6 (25%)	10 (40%)	
	Suppression (≥ 3000)	42 (42%)	17 (65.4%)	13 (52%)	7 (29.2%)	5 (20%)	
	
	
ET, esotropia; XT, exotropia; Op, operation; SE, spherical equivalent; D, diopter, BCVA, best corrected visual acuity;								
LogMAR, logarithm of the minimum angle of resolution; sec/arc, second of arc; SD, standard deviation; P, probability								
*Fisher Exact Test								
‡Based on Mann–Whitney test								

**Table 3:** The mean difference of pre- and postoperative far and near deviations in convergence excess ET cases and their controls


**Factors**	**ET' > ET ≥10pd**		***P*-value‡**
	**Slanted**	**Augmented**	
N	26	25	
Pre-op. difference	Mean ± SD	16.46 ± 5.97	13.56 ± 4.4	0.074
	Median (range)	15 (9 to 35)	10 (10 to 20)	
Post-op. difference	Mean ± SD	3.81 ± 3.54	4.92 ± 4.04	0.275
	Median (range)	4 (0 to 12)	6 (0 to 13)	
Reduction	Mean ± SD	–12.65 ± 6.16	–8.64 ± 6.1	0.014
	Median (range)	–12 (–35 to –5)	–8 (–20 to 2)	
Dose response	Mean ± SD	–6.29 ± 3.38	–5.99 ± 3.49	
	Median (range)	–5.21 (–16 to –1.6)	–7 (–10 to 2)	
Success rate (%)**		58%	28%	
	
	
ET, far esotropia; ET', near esotropia; pd, prism diopter; N, number; Op, operation;				
SD, standard deviation; P, probability				
‡Based on Mann–Whitney test				
**Success rate was defined as the postoperative far and near deviations less than 10 pd, which was stricter compared with the consideration of < 10 pd difference between the far and near deviations				

**Table 4:** The mean difference of pre- and postoperative far and near deviations in both convergence insufficiency and divergence excess exotropic cases and their controls


**Factors**	**Total**			**XT' > XT ≥ 10pd (CI)**			**XT > XT' ≥ 10pd (DE)**		
	**Slanted**	**Augmented**	***P*** **‡**	**Slanted**	**Augmented**	***P*** **‡**	**Slanted**	**Augmented**	***P*** **‡**
N	24	25	19	10	5	15	
Preop. difference	Mean ± SD	11.75 ± 3.29	11 ± 2.5	0.441	11.37 ± 2.79	11 ± 2.11	0.713	13.2 ± 4.87	11 ± 2.8	0.688
	Median (range)	10 (9 to 20)	10 (10 to 20)	10 (10 to 20)	10 (10 to 15)	10 (9 to 19)	10 (10 to 20)	
Post-op. difference	Mean ± SD	3.29 ± 2.65	2.4 ± 2.94	0.163	3.42 ± 2.69	1.2 ± 1.69	0.026	2.8 ± 2.68	3.2 ± 3.36	0.928
	Median (range)	3 (0 to 8)	2 (0 to 10)	2 (0 to 8)	0 (0 to 4)	4 (0 to 6)	2 (0 to 10)	
Reduction	Mean ± SD	–8.46 ± 3.65	–8.6 ± 3.86	0.753	–7.95 ± 2.63	–9.8 ± 3.16	0.207	–10.4 ± 6.27	–7.8 ± 4.18	0.479
	Median (range)	–8.5 (–19 to –2)	–8 (–15 to 0)	–8 (–12 to –2)	–10 (–15 to –6)	–10 (–19 to –4)	–8 (–15 to 0)	
Dose response	Mean ± SD	–7.83 ± 5.54	–7.72 ± 2.85	–7.55 ± 5.18	–8.8 ± 1.69	–8.87 ± 7.34	–7 ± 3.27	
	Median (range)	–5.75 (–20 to –1)	–8 (–10 to 0)	–6 (–20 to –1)	–10 (–10 to –6)	–5 (–19 to –1.33)	–8 (–10 to 0)	
Success rate (%)		92%	92%	89%	100%	100%	87%	
XT, exotropia; CI, convergence insufficiency; DE, divergence excess; pd, prism diopter; N, number; Op., operation; SD, standard deviation; P, probability ‡Based on Mann–Whitney test										

**Figure 1:**
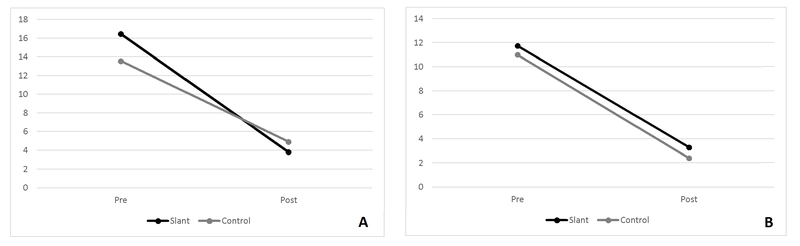
Pre- and postoperative difference between far and near deviation of both slanted and augmented groups in esotropic (A) and exotropic (B) patients.

##  DISCUSSION

###  Esotropic Patients

There was a significant difference between slant and recession methods regarding the reduction of difference between far and near deviations, but less success rates of 58% and 28% was found in the slanted and augmented recession groups, respectively. Bayramalar et al,^[2]^ Ahadzadegan et al,^[16]^ and Gharebaghi et al^[17]^ reported a success rate of 87.6%, 69%, and 70% three and six months after surgery, respectively, in small case series with esotropia and high AC/A.

Khalifi et al conducted a study^[10]^ on CE ET patients by augmented and slanted recession as well as the PF method. They found no difference in reduction among them but, like previous studies, the small sample size was a limitation.

Ellis et al studied medical records of 131 CE ET cases with a mean surgical age of nine years old.^[9]^ The reduction was 14.23 pd in slanted recession (*n* = 27), 13 pd in PF (*n* = 22), 6.8 pd in augmented recession (*n* = 58), and 5.3 pd in standard MRRec (*n* = 73). They concluded that the slanted recession caused more reduction compared to other groups. They also observed greater reduction with longer follow-ups.

According to the literature, the reported success rate of the slanted technique was between 69% and 87%, and for the augmented method, the corresponding values were 55% and 66%.^[1,2][7][8][9][10]^ We had lower success for both our slanted and augmented methods. This discrepancy could be attributed to the differences in sample size, study design, duration of follow-up, and surgical value in both augmented and slanted groups in various studies.

The amount of slanted recession is often decided based on distance and near disparity and shows a small difference in the literature.^[2,9]^ While greater discrepancy was found for augmented recession method resulting from different surgical options based on the surgeon's priority such as: (1) 1.0 to 1.5 mm more recession, (2) recession for mean angle of distance and near deviations, and (3) 1.0 mm more recession for each 10 pd difference between distance and near deviations.

In our study, the mean slanted recession was 2.38 ± 1.33 mm, while the augmented recession was 1.12 mm for ET and 1.02 mm for XT patients. It could be possible to find a non-significant difference in the success rate of two groups if augmented recession was conducted 1.0 mm for each 10 pd difference between distance and near deviations.

Various hypotheses have been suggested to justify the mechanical effect of the slanted method such as separate innervation of the superior and inferior parts of the medial rectus muscle, and different muscle length in up and down gazes compared to the primary position.^[21]^

Although van der Meulen-Schot^[18]^ and Bietti^[19]^ performed slanted recession of bimedial rectus muscles for A–V pattern in opposite directions and reduction of distance and near difference (and both achieved accepted results), Kushner^[20]^ concluded that correction of A–V pattern is achieved only by recession itself and not by slanting. Kushner believed that the effect of slanted recession was neutralized after several weeks by remodeling of the muscular sarcomeres. However, an A–V pattern reduction, of 12 % and 14 % respectively, was observed in ET and XT patients after the slant recession in our study.

###  Exotropic Patients

There was not a significant difference in reduction between bilateral lateral rectus in the slanted and augmented recession groups. In addition, the postoperative success rate of 92% was obtained for XT patients.

Our findings are in line with a study by Chun et al^[1]^ with a success rate of 84% in 31 CI XT children after six-months follow-up, and 1.0 mm slanted recession resulted in an 8.7 pd reduction.

Snir et al^[22]^ studied 18 CI adult XT patients to compare slanted and standard recession of the lateral rectus muscle. The success rate was 92% in slanted cases and 100% for distance deviation of the standard group, while the near deviation was not reduced to less than 8 pd. The dose response was 4.6 pd for the slanted group.

On the other hand, a study by Yang et al^[23]^ with 44 CI XT patients yielded a success rate of 60% in augmented recession (1.0 mm more recession) and 100% with the new R & R method, respectively (MRRes for near and LRRec for distance deviation). Their success was less in the augmented controls compared with the present study (92%).

Based on a literature review, there are limited numbers of studies about the surgical results of the slanted procedure on XT patients with CI, and we found no published article investigating the result of the slanted LRRec procedure on XT patients with true DE or XT patients with high AC/A. All publications suggested to operate for distance deviation and correct near esotropia by prescription of bifocal glasses. The present study may be the first trial to investigate the slanted procedure on true DE XT patients.

Appropriate sample size, consideration of the independent control group, and random sampling are the strong points of the present study. However, lack of comparison with other surgical techniques including PF is a limitation in the study of ET patients. Additionally, lack of equal distribution of patients in the slanted and augmented subgroups and lack of comparison with other surgical techniques including the new R & R method (MR Res. for near and LR Rec. for distance deviation) can be considered as the limitations in the study of XT patients.

In conclusion, slanted recession is recommended for CE ET patients. In XT patients, either slanted or augmented recession could be chosen according to the priority and experience of the surgeon.

##  Financial Support and Sponsorship

Nil.

##  Conflicts of Interest

There is no conflict of interest.

## References

[1] Chun Bo Young, Kang Kyung Min (2015). Early Results of Slanted Recession of the Lateral Rectus Muscle for Intermittent Exotropia with Convergence Insufficiency. *Journal of Ophthalmology*.

[2] Bayramlar H., Ünlü C., Dag Y. (2014). Slanted Medial Rectus Recession Is Effective in the Treatment of Convergence Excess Esotropia. *Journal of Pediatric Ophthalmology & Strabismus*.

[3] Griffin Grisham JD. (2007). Binocular anomalies: diagnosis and vision therapy. *Grisham JD. Binocular anomalies: diagnosis and vision therapy*.

[4] Kushner B. J., Morton G. V. (1998). Distance/near differences in intermittent exotropia. *JAMA Ophtalmology*.

[5] Hatt S., Gnanaraj L. (2006). Interventions for intermittent exotropia. *Cochrane Database of Systematic Reviews*.

[6] Birnbaum MH., Soden R., Cohen AH. (1999). Efficacy insufficiency an adult male population. *J Am Optom Assoc*.

[7] Akbari MR., Masoomian B., Jafari AK., Fard MA., Ameri A., Sadeghi AM. (2013). Slanted for insufficiency strabismus: a results in 15 cases. Binocul Vis Strabolog Q Simms Romano. *Binocul Vis Strabolog Q Simms Romano*.

[8] Raab E. L., Parks M. M. (1975). Recession of the lateral recti. Effect of preoperative fusion and distance-near relationship. *JAMA Ophtalmology*.

[9] Ellis G. S., Pritchard C. H., Baham L., Babiuch A. (2017). Medial Rectus Surgery for Convergence Excess Esotropia with an Accommodative Component: A Comparison of Augmented Recession, Slanted Recession, and Recession with Posterior Fixation. *American Orthoptic Journal*.

[10] Khalifa Y. M. (2011). Augmented medial rectus recession, medial rectus recession plus Faden, and slanted medial rectus recession for convergence excess esotropia.. *European Journal of Ophthalmology*.

[11] West CE., Repka MX. (1994). A comparison of surgical techniques for the treatment of acquired esotropia with increased accommodative convergence/accommodation ratio. J Pediatr Ophthalmol. *Repka MX. A comparison of surgical techniques for the treatment of acquired esotropia with increased accommodative convergence/accommodation ratio. J Pediatr Ophthalmol Strabismus*.

[12] Yam J. C. S., Wu P. K. W., Chong G. S. L., Wong U. S. F., Chan C. W. N., Ko S. T. C. (2012). Long-term ocular alignment after bilateral lateral rectus recession in children with infantile and intermittent exotropia. *Journal of American Association for Pediatric Ophthalmology and Strabismus*.

[13] Rajavi Z., Lashgari A., Sabbaghi H., Behradfar N., Yaseri M. (2017). The incidence of reoperation and related risk factors among patients with infantile exotropia. *Journal of Pediatric Ophthalmology and Strabismus*.

[14] Kushner BJ. Strabismus.

[15] Parks MM., Tasman W., Jaeger EA. (1998). Extraocular muscles. *Duanes clinical ophthalmology, revised edition*.

[16] Ahadzadegan I. (2001). Slanted recession of medial recti to treat convergence excess esotropia. *Iran J Ophthalmol*.

[17] Gharabaghi D., Zanjani LK. (2006). Faden procedure, and slanted the treatment of high accommodative convergence/accommodation ratio esotropia. J Pediatr Ophthalmol Strabismus. *Zanjani LK. Comparison of results of medial rectus muscle recession using augmentation*.

[18] van der Meulen-Schot H. M., van der Meulen S. B., Simonsz H. J. (2008). Caudal or cranial partial tenotomy of the horizontal rectus muscles in A and V pattern strabismus. *British Journal of Ophthalmology*.

[19] Bietti GB.

[20] Kushner B. J. (2011). Insertion slanting strabismus surgical procedures. *JAMA Ophtalmology*.

[21] da Silva Costa R. M., Kung J., Poukens V., Yoo L., Tychsen L., Demer J. L. (2011). Intramuscular Innervation of Primate Extraocular Muscles: Unique Compartmentalization in Horizontal Recti. *Investigative Opthalmology & Visual Science*.

[22] Snir M., Axer-Siegel R., Shalev B., Sherf I., Yassur Y. (1999). Slanted lateral rectus recession for exotropia with convergence weakness. *Ophthalmology*.

[23] Yang H. K., Hwang J. (2011). Surgical Outcomes in Convergence Insufficiency-Type Exotropia. *Ophthalmology*.

